# The Persistence of Self-injurious and Aggressive Behavior in Males with Fragile X Syndrome Over 8 Years: A Longitudinal Study of Prevalence and Predictive Risk Markers

**DOI:** 10.1007/s10803-019-04002-3

**Published:** 2019-04-24

**Authors:** Hayley Crawford, Efthalia Karakatsani, Gursharan Singla, Chris Oliver

**Affiliations:** 1 Centre for Innovative Research Across the Life Course, Coventry University, Coventry, UK; 2 Cerebra Centre for Neurodevelopmental Disorders, School of Psychology, University of Birmingham, 52 Pritchatts Road, Birmingham, B15 2TT UK; 3 Department of Psychology, University of Wolverhampton, Wolverhampton, UK

**Keywords:** Fragile X syndrome, Self-injurious behavior, Aggression, Repetitive behavior, Impulsivity, Autism, Risk markers, Challenging behavior, Early intervention

## Abstract

Self-injurious and aggressive behaviors are common in fragile X syndrome (FXS). However, little is known about the persistence of these behaviors and associated risk markers. We established the prevalence and persistence of self-injurious and aggressive behaviors over eight years in males with FXS, and associations with risk markers. Results showed 77% and 69% persistence rates for self-injurious and aggressive behavior, respectively. Baseline levels of repetitive behavior predicted persistent self-injurious behavior. Chronological age, impulsivity and overactivity were associated with persistent aggressive behavior but only impulsivity *predicted* persistence. This is the first study to document the persistence of self-injurious and aggressive behavior in FXS over the medium to long term and to identify behavioral risk markers that might facilitate targeted early intervention.

## Introduction

Self-injury and aggression are common and detrimental behaviors associated with intellectual disability (ID). Prevalence rates for self-injurious behavior in people with ID of heterogeneous aetiology range from 4 to 17% (Collacott et al. 1998; Cooper et al. 2009; Oliver et al. 1987) whilst rates for aggressive behavior range from 7 to 25% (Cooper et al. 2009; Emerson et al. 2001). Compared to those with ID of heterogeneous aetiology, prevalence rates of self-injury and aggression are elevated in some genetic syndromes. For example, individuals with Cornelia de Lange, Cri du Chat, fragile X (FXS), Lowe, Prader-Willi and Smith-Magenis syndromes are significantly more likely to display self-injury than a contrast group of individuals with ID of heterogeneous aetiology (Arron et al. 2011).

Establishing the *persistence* of self-injurious and aggressive behavior across the lifespan is important for characterising the natural history of these behaviors and informing intervention strategies. In individuals with ID, self-injurious and aggressive behaviors typically emerge during childhood and prevalence rates then increase with age into adulthood and decrease thereafter (Davies and Oliver 2013). Additionally, high rates of persistence of self-injurious and aggressive behavior are evident in individuals with ID. Longitudinal studies of self-injury report 58% persistence over 2 years (Davies and Oliver 2016), 71% persistence over 7 years (Emerson et al. 2001), and 84% persistence over 20 years (Taylor et al. 2011). Persistence of aggressive behavior has been reported in children with ID over a 2-year period (69%; Davies and Oliver 2016). Three-year persistence of self-injurious and aggressive behavior has been investigated in specific neurodevelopmental disorders with 84% persistence of self-injury and 67% persistence of aggressive behavior reported in individuals with tuberous sclerosis complex (Wilde et al. 2018), and 78% persistence of self-injury in individuals with autism spectrum disorder (ASD; Richards et al. 2016). Taken together, this research suggests that the prevalence of self-injurious and aggressive behavior increases with age into adulthood and is highly persistent both in individuals with ID of heterogeneous aetiology and in those with neurodevelopmental disorders. As such, the importance of early intervention strategies has been highlighted (see Davies and Oliver 2016; Oliver and Richards 2015).

The presence of self-injurious and aggressive behavior in individuals with ID has been associated with specific behavioral characteristics with emerging evidence that these behavioral characteristics are also associated with the *persistence* of self-injurious and aggressive behavior. For example, severity of ID is associated with the presence of self-injurious and aggressive behavior in heterogeneous ID samples (Cooper et al. 2009; McClintock et al. 2003), and in some neurodevelopmental disorders such as Cornelia de Lange syndrome, although this is not the case in other genetic syndromes including FXS (Arron et al. 2011; Symons et al. 2003). In addition, the presence of some genetic syndromes is associated with an increased risk of displaying self-injurious and aggressive behavior, over and above that accounted for by level of ID (Arron et al. 2011). When level of ID is controlled for, the prevalence of self-injury is also heightened in individuals with ASD (Richards et al. 2012) and in those with a number of genetic syndromes who score higher on a screening measure for ASD (Arron et al. 2011). More specifically, repetitive behavior is associated with a threefold increase of aggressive behavior and a sixfold increase of self-injury (Oliver et al. 2012).

In addition to ASD, characteristics associated with attention-deficit-hyperactivity disorder (ADHD) have also been associated with heightened prevalence and persistence of self-injurious and aggressive behavior. For example, higher levels of impulsivity have been associated with heightened prevalence of self-injurious and aggressive behavior in a number of genetic syndromes including FXS (Arron et al. 2011) and in idiopathic ID (Rojahn et al. 2004). Impulsivity has also been associated with persistence of self-injury in individuals with ASD (Richards et al. 2016) and with persistence of both self-injurious and aggressive behavior in individuals with tuberous sclerosis complex (Wilde et al. 2018). In heterogeneous ID, ADHD is associated with increased persistence of aggressive behavior over time (Cooper et al. 2009). Taken together, this research suggests that ASD and ADHD characteristics confer heightened risk for the presence and persistence of self-injurious and aggressive behavior. Importantly, characteristics of ASD and ADHD are elevated in some neurodevelopmental disorders, such as FXS, and therefore such groups are likely to be at high risk for persistent self-injurious and aggressive behavior.

### Fragile X Syndrome: A High Risk Population

FXS occurs in approximately 1 in 4000 males and 1 in 8000 females (Crawford et al. 2001). It is caused by an expansion of cytosine-guanine-guanine (CGG) repeats (> 200) on the *FMR1* gene, which results in gene methylation and a subsequent reduction of FMRP. As an X-linked disorder, males and females are differentially affected. Due to phenotypic gender differences, the present study reports data for males with FXS only. Alongside moderate ID, self-injurious and aggressive behavior is phenotypic of FXS, with 54–58% of individuals reported to self-injure at some point during their lifetime (Richards et al. 2012; Symons et al. 2003) and 50% reported to display aggressive behavior (Arron et al. 2011).

Hand biting is reported to be the most common form of self-injury in FXS (Richards et al. 2012; Symons et al. 2003), occurring in 26% of males, and changes to routine and the presentation of difficult demands have been cited as the most common causes of self-injurious behavior in FXS (Symons et al. 2003). The most common forms of aggressive behaviors have been reported as hitting others (49%) and kicking others (30%; Hessl et al. 2008). There is evidence that self-injury may be persistent over the short term in individuals with FXS, with 81% of participants who manifest self-injurious behavior continuing to engage in this behavior 1 month from the original report (Symons et al. 2003). Similarly, aggressive behavior in FXS may be persistent over a three-year time period as indicated by stable ‘problem behavior’, a broader construct including aggressive behavior (Hatton et al. 2002). Considering the relationship between age and self-injurious and aggressive behavior (Davies and Oliver 2013), it is important that the persistence of self-injurious and aggressive behaviors is investigated across a wide age range and longer time frame.

FXS is associated with behavioral characteristics that have previously been associated with self-injurious and aggressive behavior. Specifically, overactivity and impulsivity, as well as repetitive behavior and impairments in social communication are heightened in FXS (Baumgardner et al. 1995; Farzin et al. 2006; Hatton et al. 2002; Munir et al. 2000; Smith et al. 2012). These behavioral features contribute to the elevated prevalence rates of both ADHD and ASD in individuals with FXS. ADHD occurs in 53–73% of individuals with FXS (Baumgardner et al. 1995; Sullivan et al. 2006), which is higher than the prevalence rate of 33% in age and IQ-matched peers (Baumgardner et al. 1995) and 5% in the general population (American Psychiatric Association 2013). Similarly, the presence of ASD is also elevated with a 30% prevalence rate in FXS (Richards et al. 2015) compared to 1% of the general population (Baird et al. 2006). The current literature suggests that many behaviors associated with both ADHD and ASD remain largely stable over time in individuals with FXS (Crawford et al. 2018; Hatton et al. 2002; McDuffie et al. 2010; Thurman et al. 2015). However, inconsistent results have also been reported regarding the persistence of some social behaviors associated with ASD with both subtle improvements (McDuffie et al. 2010) and increasing impairment (Thurman et al. 2015) being reported with age. In addition, our recent research demonstrated an improvement in impulsivity and repetitive questioning in males with FXS who did not have elevated autism symptoms (Crawford et al. 2018).

Given the high prevalence of ASD and ADHD related behaviors in FXS, and the strong association between these behaviors and self-injurious and aggressive behaviors, it is important that this area of research is extended by investigating the persistence of self-injurious and aggressive behavior, and the characteristics associated with persistence, using longitudinal data. Delineating the developmental trajectories of such associations may help in identifying potential early behavioral risk markers for self-injurious and aggressive behavior in young populations with FXS and may also inform strategic early and targeted interventions that increase quality of life.

Studying the phenomenology of ASD and ADHD in relation to self-injurious and aggressive behavior, as opposed to studying diagnostic categories, goes some way towards overcoming with the challenge of diagnostic determination and specificity caused by the overlap of behavioral features associated with FXS, ASD and ADHD (e.g. social impairment), and with the subtle but important differences between those with FXS, and idiopathic ASD (see Abbeduto et al. 2014 for a review).

The current study will employ longitudinal analyses of data collected over an eight-year time period to address three aims:

1. To investigate the prevalence and persistence of self-injury and aggression in individuals with FXS over an eight-year time period.
2. To delineate the association between impairments in social communication, repetitive behavior, overactivity, and impulsivity, and the persistence of self-injurious and aggressive behavior in individuals with FXS.
3. To investigate which associated behavioral characteristics can *predict* the persistence of self-injurious and aggressive behavior in individuals with FXS.

## Method

### Recruitment

This study was conducted as part of a large-scale questionnaire study investigating the behavioral phenotypes of a range of different neurodevelopmental disorders. Participants were recruited over three time points. At Time 1 (T_1_; 2003–2004), parents and carers of 762 males with FXS were contacted through the Fragile X Society, the UK family support group, and asked if they would like to participate in the study. Of these prospective participants, 211 (27%) participated. At Time 2 (T_2_; 2006–2007) and Time 3 (T_3_; 2011–2012), parents and carers of all 211 males with FXS who participated at T_1_ were contacted directly and invited to participate in the follow-up stages of the study. Of the 211 participants, 148 (70%) took part at T_2_ and 91 (43%) took part at T_3_. Data from questionnaires were included in the present study providing they contained information regarding diagnosis, and age or date of birth, and had missing data on no more than 25% of the 14 measures in the questionnaire pack at any time point. After applying the exclusion criteria, data were available on 79 males with FXS.

### Participants

Demographic characteristics of the 79 participants are presented in Table 1. Descriptive analysis showed that the majority of participants were verbal (able to speak or sign more than 30 words), had a normal range of hearing and vision abilities, were mobile, and demonstrated self-help skills categorised as ‘partly able’ or ‘able’, based on the Wessex scale (Kushlick et al. 1973). Twenty-seven (34%) of the participants took part in a follow up study where their FXS diagnoses were confirmed through genetic testing. The remaining participants’ FXS diagnoses were confirmed through parental report of a diagnosis from a relevant professional (GP, paediatrician, or clinical geneticist).

**Table 1:** Participant characteristics

		Time 1 (n = 79)	Time 2 (n = 79)	Time 3 (n = 79)
Age in years	Mean (SD)	17.64 (9.21)	20.22 (9.15)	25.01 (9.00)
	Range	6–47	9–49	14–54
Gender	% male	100	100	100
Wessex self-help	% partly able/able	84.8	89.9	91.1
Mobility^a^	% mobile	98.7	97.5	98.7
Speech^b^	% verbal	87.3	89.9	92.4
Wessex vision	% normal	92.4	94.9	89.9
Wessex hearing	% normal	93.7	93.7	97.5
SCQ total score	Mean (SD)	20.80 (5.96)	20.18 (6.28)	18.39 (5.95)
	Range	6–34	6–35	7–33
SCQ score (ASD cut off)	% score above 15	75.9	69.6	65.8
TAQ total score	Mean (SD)	38.05 (19.66)	34.06 (19.86)	33.87 (19.65)
	Range	1–72	0–71	0–71

^a^Able to walk unaided

^b^Speak/sign more than 30 words

### Measures

The following informant measures were used:

### Adaptive Behavior: The Wessex Scale

The Wessex scale (Kushlick et al. 1973) was developed to assess the social and physical capabilities of children and adults with ID. It comprises five subscales, including: continence, mobility, self-help skills, speech and literacy. For the present study, the self-help subscale was used as an estimate of the degree of adaptive behavior ability. The Wessex scale is an effective tool for large-scale postal questionnaire studies and has modest inter-rater reliability at subscale level for both children and adults with ID with mean reliability coefficients of 0.62 for overall classification and 0.54 for item-level reliability (Palmer and Jenkins 1982).

### Self-Injurious and Aggressive Behavior: The Challenging Behaviour Questionnaire

The Challenging Behaviour Questionnaire (CBQ; Hyman et al. 2002) was developed to evaluate the presence of self-injury, physical aggression, destruction of property, and stereotyped behavior occurring in the last month. The measure also examines eight topographies of self-injury that were adapted from Bodfish et al. (1995). For the present study items evaluating the presence of self-injurious and aggressive behavior were used. The CBQ demonstrates good inter-rater reliability with reliability coefficients ranging from 0.61 to 0.89 (Hyman et al. 2002).

### Attention-Deficit Hyperactivity Disorder Characteristics: The Activity Questionnaire

The Activity Questionnaire (TAQ; Burbidge et al. 2010) was developed to assess behaviors indicative of overactivity and impulsivity, which are associated with attention-deficit hyperactivity disorder, in individuals with ID. The TAQ has 18 items which form three subscales of overactivity, impulsivity and impulsive speech. Internal consistency for the subscales is good with alpha coefficients ranging from 0.67 to 0.94. Inter-rater and test–retest reliability indices for subscales and total score exceed 0.70. Item level inter-rater reliability ranges from 0.31 to 0.75 (mean 0.56) and test–retest reliability ranges from 0.60 to 0.90 (mean 0.75).

### Autism Characteristics: The Social Communication Questionnaire

The Social Communication Questionnaire (SCQ; Rutter et al. 2003) is a 40-item questionnaire designed to assess characteristics associated with ASD. There are three subscales: social interaction, communication and repetitive behavior. Each item on the questionnaire describes a specific social, communicative or repetitive behavior and requires a Yes/No response to indicate the presence or absence of each behavior. A score of 22 or more constitutes the cut-off score for possible autism, with a score of 15 or more indicating possible ASD. In terms of convergent validity, significant correlations have been observed between the Autism Diagnostic Observation Schedule (Lord et al. 1999) and the Autism Diagnostic Interview (Le Couteur et al. 1989). Sensitivity and specificity of 0.85 and 0.85, respectively, are reported for the cut-off for possible ASD, and values of 0.75 and 0.60 are reported for the cut-off for autism. Internal consistency is good (α = 0.90 for the total scale). At T1, the Lifetime Version of the SCQ was employed. At T_2_ and T_3_, the Current Version was administered as this is recommended to evaluate change over time.

### Procedure

At each time point, parents and carers of prospective participants were mailed a covering letter, an information sheet about the study, a questionnaire pack consisting of a number of measures, and a consent form. Measures were counterbalanced to mitigate order effects. Data from all time points for the measures included in the present study (Wessex for sample characterisation, and CBQ, TAQ and SCQ) were extracted for analysis.

### Data Analysis

Data were tested for normality using Kolmogorov–Smirnov tests. Where data did not meet criteria for a normal distribution (*p* < 0.05) non-parametric tests were employed.

To investigate the persistence of self-injurious and aggressive behavior in individuals with FXS, descriptive analyses of the CBQ were conducted at T_1_, T_2_, and T_3_. For analyses concerning self-injury, participants were divided into three subgroups: (a) those that show *persistent* self-injury (present at T_1_, T_2_ and T_3_), (b) those that show *transient* self-injury (present at T_1_, T_2_, *or* T_3_ but not present for all of those time points), and (c) those that show an *absence* of self-injury (absent at T_1_, T_2_ and T_3_). For analyses regarding aggressive behavior, participants were divided into three subgroups using the same criteria.

To investigate the demographic and behavioral characteristics associated with persistence of self-injurious and aggressive behavior, a series of Kruskal–Wallis tests were conducted to assess differences between each of the sub-groups on subscale scores of impulsivity and overactivity (using the TAQ), and on subscale scores of social interaction, communication, and repetitive behavior (using the SCQ). Differences between the subgroups on the demographic characteristics of chronological age and adaptive behavior ability, as measured by the Wessex, were also explored. Where between-group differences were revealed, the source of difference was explored using Mann–Whitney *U* tests. Binary logistic regressions were then undertaken to investigate whether the demographic and behavioral characteristics associated with persistence of self-injurious or aggressive behavior *predicted* the persistence of behavior.

## Results

### Prevalence and Persistence of Self-injurious and Aggressive Behavior in FXS

The first aim of the study was to investigate the prevalence and persistence of self-injurious and aggressive behavior in males with FXS. Self-injurious behavior was demonstrated by 49.4% of participants at each time point. There was more fluctuation in the prevalence rates of aggressive behavior, which was demonstrated by 40.5, 31.6, and 36.7% of participants at T_1_, T_2_, and T_3_, respectively. The number of participants showing absent, transient and persistent self-injurious and aggressive behavior across the eight-year time period is shown in Table 2. The findings show that 34.2% and 21.5% of the total sample displayed persistent self-injurious and aggressive behavior, respectively. Overall, 76.9% of participants who displayed self-injury at T_1_ continued to demonstrate this behavior at T_3_ whereas 68.8% of participants who displayed aggressive behavior at T_1_ continued to display this behavior at T_3_.

**Table 2:** Number of participants and percentages (in parentheses) showing absent, transient and persistent self-injurious and aggressive behavior between Time 1 and Time 3

	Total sample (n = 79)			Participants with behavior at T_1_ (n = 39)	
Behavior	Absent	Transient	Persistent	Persistence at T_2_	Persistence at T_3_
Self-injury (%)	25 (31.6)	27 (34.2)	27 (34.2)	30 (76.9)	30 (76.9)

	Total sample (n = 79)			Participants with behavior at T1 (n = 32)	
Behavior	Absent	Transient	Persistent	Persistence at T_2_	Persistence at T_3_
Aggression (%)	36 (45.6)	26 (32.9)	17 (21.5)	19 (59.4)	22 (68.8)

### Demographic and Behavioral Characteristics Associated with the Persistence of Self-injurious and Aggressive Behavior in Individuals with FXS

The second aim of the study was to investigate the demographic and behavioral characteristics associated with the persistence of self-injurious and aggressive behavior in individuals with FXS. Kruskal–Wallis tests revealed that participants who never displayed self-injury, participants who showed persistent self-injury, and participants who showed transient self-injury differed significantly in terms of T_1_ repetitive behavior only (χ^2^ (2) = 8.396, *p* = 0.015). Mann–Whitney *U* tests confirmed that this difference was driven by lower repetitive behavior scores in those with absent self-injury compared to those that showed persistent (*U* = 186.500, *p* = 0.005) or transient self-injury (*U* = 226.500; *p* = 0.040). The results from this analysis are shown in Table 3.

**Table 3:** Demographic and behavioral data of subgroups of participants with FXS showing either persistent, transient, or absent self-injurious behavior

		Persistent	Transient	Absent	Kruskal–Wallis	df	*p*	Post hoc Mann–Whitney *U*
N		27	27	25				
Age	Mean (SD)	15.38 (7.67)	19.08 (9.79)	18.53 (9.97)	2.781	2	0.249	
Wessex self-help score	Mean (SD)	7.06 (1.48)	7.11 (1.60)	7.84 (1.46)	4.723	2	0.094	
TAQ impulsivity	Mean (SD)	17.81 (6.74)	16.33 (7.07)	12.90 (7.94)	5.794	2	0.055	
TAQ overactivity	Mean (SD)	20.63 (11.40)	17.97 (17.97)	14.92 (10.54)	3.768	2	0.152	
SCQ communication	Mean (SD)	7.37 (1.60)	7.16 (2.89)	7.38 (2.37)	0.076	2	0.963	
SCQ social interaction	Mean (SD)	9.28 (2.49)	7.57 (3.39)	8.18 (3.19)	2.910	2	0.233	
SCQ repetitive behavior	Mean (SD)	5.11 (1.74)	4.63 (1.80)	3.36 (2.22)	8.396	2	**0.015**	Persistent > absent; transient > absent

Bold values is statistically significant (*p* < .05)

**Fig. 1:**
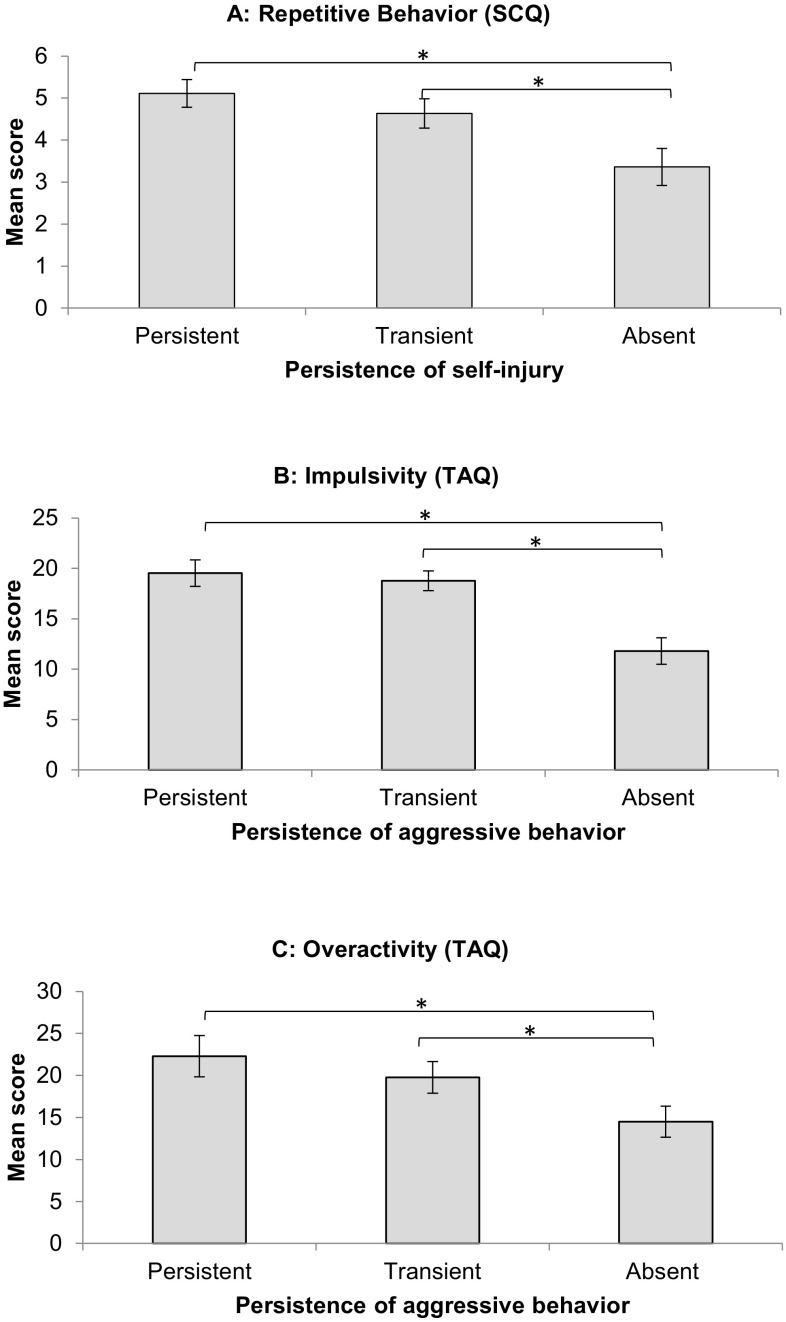
Differences between those showing persistent, transient or absent self-injury on the baseline measure of repetitive behavior (**a**). Differences between those showing persistent, transient or absent aggressive behavior on baseline measures of impulsivity (**b**) and overactivity (**c**). Error bars represent standard error

Kruskal–Wallis tests also revealed that participants who showed either persistent, absent or transient aggressive behavior differed significantly in chronological age (χ^2^ (2) = 9.481, *p* = 0.009), impulsivity (χ^2^ (2) = 17.422, *p* < 0.001) and in overactivity (χ^2^ (2) 7.580, *p* = 0.023). Participants with persistent aggressive behavior were significantly younger at T_1_ than those with transient (*U* = 135.000, *p* = 0.033) or absent aggressive behavior (*U* = 150.000, *p* = 0.003). In addition, participants who showed absent aggressive behavior scored significantly lower on measures of impulsivity at T_1_ than those that showed persistent (*U* = 125.000, *p* = 0.001) or transient aggressive behavior (*U* = 231.000, *p* = 0.001). Finally, participants who showed absent aggressive behavior scored significantly lower on measures of overactivity at T_1_ than those that showed persistent (*U* = 176.500, *p* = 0.014) or transient aggressive behavior (*U* = 329.500, *p* = 0.048). These results are shown in Table 4. Figure 1 shows the mean scores for the measures that significantly differentiate each of the sub-groups.

**Table 4:** Demographic and behavioral data of the subgroups of participants with FXS showing either persistent, transient or absent aggressive behavior

		Persistent	Transient	Absent	Kruskal–Wallis	df	*p*	Post hoc (Mann–Whitney U)
N		17	26	36				
Age	Mean (SD)	13.01 (7.03)	17.35 (8.63)	20.05 (9.85)	**9.481**	**2**	**0.009**	Absent > persistent; persistent < transient
Wessex self-help score	Mean (SD)	6.79 (1.40)	7.35 (1.35)	7.56 (1.70)	3.955	2	0.138	
TAQ impulsivity	Mean (SD)	19.53 (5.44)	18.77 (5.06)	11.79 (7.86)	**17.422**	**2**	**<0.001**	Absent < persistent; absent < transient
TAQ over-activity	Mean (SD)	22.29 (10.15)	19.77 (9.58)	14.50 (11.02)	**7.580**	**2**	**0.023**	Absent < persistent; absent < transient
SCQ communication	Mean (SD)	7.22 (1.73)	7.31 (2.47)	7.33 (2.51)	0.161	2	0.923	
SCQ social interaction	Mean (SD)	8.11 (2.39)	8.64 (3.40)	8.21 (3.20)	0.822	2	0.663	
SCQ repetitive behavior	Mean (SD)	4.59 (2.09)	5.00 (1.81)	3.86 (2.07)	4.036	2	0.133	

Bold values are statistically significant (*p* < .05)

### Predicting Persistent Self-injurious and Aggressive Behavior

The third aim of the study was to evaluate which demographic and behavioral characteristics that were associated with the persistence of self-injurious or aggressive behavior *predict* the *persistence* of such behavior in individuals with FXS.

First, a binary logistic regression was performed with self-injury (persistent or absent; n = 52) as the dependent variable. Earlier analyses indicated that only repetitive behavior was shown to differentiate the subgroups of participants displaying persistent, transient or absent self-injury and this was, therefore, the only predictor variable included in the binary logistic regression. The logistic regression model (χ^2^ (1) = 9.414, *p* = 0.002) correctly classified 67.3% of cases and restricted, repetitive behavior significantly contributed to the prediction of persistent self-injury (Wald = 7.495, df (1), *p* = 0.006). Increasing scores on the measure of restricted, repetitive and stereotyped behavior was associated with an increased likelihood of exhibiting persistent self-injury (odds ratio = 1.566).

A second binary logistic regression was conducted with aggressive behavior (persistent or absent; n = 53) as the dependent variable. Predictor variables of interest were chronological age, impulsivity, and overactivity. The logistic regression model (χ^2^ (3) = 18.043, *p* < 0.001) correctly classified 75.5% of cases. Only impulsivity significantly contributed to the prediction of persistent aggressive behavior (Wald = 6.291, df (1), *p* = 0.012). Increasing impulsivity scores was associated with an increased likelihood of exhibiting persistent aggressive behavior (odds ratio = 1.265), but increasing overactivity scores and chronological age did not significantly contribute to the model (both *p* > 0.05).

## Discussion

This longitudinal study investigated the persistence of self-injurious and aggressive behavior in males with FXS over an eight-year time period. In addition, behavioral correlates associated with the persistence of self-injury and aggression were investigated. Results indicated that 77% of individuals with FXS who showed self-injury at T_1_ continued to demonstrate this behavior 8 years later whilst 69% of individuals who showed aggressive behavior still demonstrated this behavior 8 years later. Severity of restricted and repetitive behavior at T_1_ differentiated those who had never displayed self-injurious behavior from those that displayed either transient or persistent self-injurious behavior. Chronological age, impulsivity and overactivity at T_1_ was associated with persistence of aggressive behavior. Specifically, individuals with persistent aggressive behavior were older than those with absent or transient aggression. In addition, individuals with absent aggressive behavior demonstrated lower levels of impulsivity and overactivity than those with transient or persistent aggression. Finally, higher levels of restricted and repetitive behavior and higher levels of impulsivity at T1 *predicted* persistent self-injurious and aggressive behavior, respectively. Taken together, these results indicate that restricted and repetitive behavior is a risk marker for persistent self-injurious behavior whilst early impulsivity is a risk marker for persistent aggressive behavior.

Almost 50% of participants demonstrated self-injury within the preceding month at each of the three measurement time points. These prevalence rates are similar to those reported in existing literature (Arron et al. 2011; Richards et al. 2012; Symons et al. 2003) and corroborate reports that self-injury is a common characteristic of FXS. The results reported here also indicate that for individuals with FXS who show self-injury, this behavior is highly likely to persist with 77% of participants continuing to demonstrate this behavior 8 years later. This persistence rate is similar to that reported in existing literature measured over a shorter time frame. Symons et al. (2003) reported 84% persistence of self-injurious behavior 1 month after baseline measurement in individuals with FXS. The persistence rate in FXS is also broadly consistent with other populations including heterogeneous ID (71% over 7 years; Emerson et al. 2001), tuberous sclerosis complex (85% over 3 years; Wilde et al. 2018), and ASD (78% over 3 years; Richards et al. 2016).

Prevalence rates for aggressive behavior at each time point varied from 31 to 40% suggesting that this behavior is slightly less common than self-injury in males with FXS. However, as with self-injurious behavior, aggressive behavior was persistent over 8 years for the majority of participants displaying this behavior (69%). The persistence rate of aggressive behavior in FXS is similar to that of idiopathic ID where two-year persistence of 68.4% (Cooper et al. 2009) and 15–18 month persistence of 69% (Davies and Oliver 2016) has been reported. The persistent nature of self-injurious behavior and aggression underlines the need to develop early interventions that target this group of individuals who are at higher risk for the development of self-injurious and aggressive behavior.

The current study identified risk markers for persistent self-injurious behaviors. Specifically, restricted and repetitive behavior differentiated participants showing absent, transient or persistent self-injury with lower severity of repetitive behavior being associated with absent self-injurious behavior. To further support the strength of this association, early indicators of restricted and repetitive behavior were shown to *predict* the persistence of self-injurious behavior. Other ASD-related characteristics such as communication and social interaction were not significantly related to the persistence of self-injurious behavior, nor were the ADHD-related characteristics of impulsivity and overactivity. In addition, neither chronological age nor level of ability were related to the persistence of self-injury.

These results are consistent with existing literature that suggests chronological age and level of ability are not associated with the presence of self-injury in FXS (Symons et al. 2003). Impulsivity has often been associated with prevalence and persistence of self-injury in other populations (Arron et al. 2011; Richards et al. 2012, 2016; Wilde et al. 2018) but the results from the current study do not support this association in FXS (see also Davies and Oliver 2016). However, the difference in impulsivity between those showing absent, transient or persistent aggression approached significance (*p* = 0.055) and so limited statistical power in the current study may explain this discrepancy. Existing studies have highlighted the presence of ASD (Richards et al. 2012) and social impairment (Arron et al. 2011) as a risk marker for self-injurious behavior in FXS, although other studies have revealed no association (Symons et al. 2003). However, in the current study, it was only the restricted and repetitive behavior component of the ASD phenotype that was associated with persistence of self-injurious behavior. These inconsistencies with other literature may be related to the current study assessing the relationship between ASD characteristics and the *persistence* of self-injurious behavior rather than the presence or prevalence of self-injurious behavior. Impulsivity and deficits in social interaction have, however, been related to persistence of self-injury in individuals with ASD (Richards et al. 2016). This suggests that the behavioral risk markers for persistent self-injury may differ between those with FXS and those with idiopathic ASD. This is an important characterisation to add to the growing body of literature highlighting subtle differences between the FXS and ASD behavioral phenotypes (see Abbeduto et al. 2014 for a review).

The current study also identified putative risk markers for persistent aggressive behavior in males with FXS. Chronological age, impulsivity, and overactivity differentiated the subgroups of participants displaying absent, transient or persistent aggressive behavior. However, it was only the behavioral characteristic of impulsivity that *predicted* persistent aggressive behavior. Impulsivity has been consistently noted in the existing literature as a risk marker for the presence of aggressive behavior in a range of populations including idiopathic ID, and genetic syndromes including Angelman, Cri du Chat, Cornelia de Lange, FXS, Prader-Willi and Lowe syndrome (Arron et al. 2011; Davies and Oliver 2016). It has also been associated with the persistence of aggressive behavior in tuberous sclerosis complex (Wilde et al. 2018). Taken together, these results indicate that impulsivity is a particularly sensitive risk marker for the presence and persistence of aggressive behavior in neurodevelopmental disorders.

Interestingly, different risk markers were revealed for persistent self-injurious and aggressive behavior. Repetitive behavior predicted persistent self-injury whilst impulsivity predicted persistent aggressive behavior. This highlights the importance of considering self-injurious and aggressive behavior separately, rather than under the umbrella construct of challenging behavior. Davies and Oliver (2016) report similar findings in individuals with idiopathic ID where the relative risk of the cumulative incidence of self-injurious and aggressive behavior over 15–18 months was significantly increased by repetitive behavior and impulsivity, respectively. Taken together, these results point to different mechanisms underlying these two behavioral characteristics. Recent models of self-injurious behavior suggest that repetitive behavior may evolve into self-injury through social reinforcement (Oliver and Richards 2015). Contemporary accounts of impulsivity suggest that the association between impulsivity and aggressive behavior is linked to impairments in executive functioning difficulties, particularly in terms of inhibiting pre-potent behavioral responses and stopping ongoing responses (Davies and Oliver 2016; Oliver and Richards 2015). This explanatory framework may be particularly useful for considering this association in FXS as it is associated with significant deficits in inhibition and other executive functions compared to participants matched on mental age (Hooper et al. 2008).

Limitations of this study include the lack of information on potential interventions accessed by participants for self-injurious or aggressive behavior, or for the associated behavioral correlates identified in the present study. Uptake of intervention may influence the rates of persistence of self-injurious and/or aggressive behavior. Persistence of both self-injurious and aggressive behavior were high indicating little influence of potential intervention. However, it is not known whether these rates reflect the natural course of behavior or limited access to or effectiveness of intervention. The study is also limited by the sample size consisting only of males. Whilst this was a strategic decision based on the striking phenotypic differences between males and females with FXS, and increased severity of behavioral difficulties in males with FXS, future research should nonetheless include females so that the behavioral phenotype can be further understood in this under-researched population. Finally, the age range of the participants in the current study was wide and so the extent to which the results may be driven by particular age groups is unknown. Chronological age was factored into the statistical analyses and was not associated with persistence of self-injury. It was, however, associated with persistence of aggressive behavior but was not a predictive risk marker.

Despite the limitations reported above, to our knowledge, this is the first study to evaluate the persistence of self-injurious and aggressive behaviors in males with FXS longitudinally. This study uses robust measurement tools that have been designed to assess behavioral phenotypes of neurodevelopmental disorders associated with ID. The specificity of the behaviors measured is a particular strength, compared to many existing studies that group the behavioral characteristics measured individually here as ‘problem’ behaviors, or those that group self-injurious and aggressive behaviors into a broad concept of challenging behavior. The merit of this approach is indicated by revealing *different* risk markers for persistent self-injurious and aggressive behaviors. In addition, measuring such behavioral characteristics over an 8-year time period offers important insight into the natural history of clinically significant behaviors and associated risk markers.

In summary, the results reported here have identified that self-injurious and aggressive behavior are persistent features of the FXS phenotype. In addition, risk markers of repetitive behavior and impulsivity are associated with persistent self-injury and aggression, respectively, over time. Given the high rates of both ASD and ADHD in FXS, the role of repetitive behavior and impulsivity as risk markers is particularly important. The results reported here could facilitate early intervention strategies by targeting individuals who are at the highest risk of persistent self-injury and aggression with an aim of reducing the likelihood of persistence or even prevent the emergence of behavior.

## References

[CR1] Abbeduto L, McDuffie A, Thurman AJ (2014). The fragile X syndrome-autism comorbidity: What do we really know?. Frontiers in Genetics.

[CR2] American Psychiatric Association (2013). Diagnostic and statistical manual of mental disorders.

[CR3] Arron K, Oliver C, Moss J, Berg K, Burbidge C (2011). The prevalence and phenomenology of self-injurious and aggressive behaviour in genetic syndromes. Journal of Intellectual Disability Research.

[CR4] Baird G, Simonoff E, Pickles A, Chandler S, Loucas T, Meldrum D, Charman T (2006). Prevalence of disorders of the autism spectrum in a population cohort of children in South Thames: The special needs and autism project (SNAP). The lancet.

[CR5] Baumgardner TL, Reiss AL, Freund LS, Abrams MT (1995). Specification of the neurobehavioral phenotype in males with fragile X syndrome. Pediatrics.

[CR6] Bodfish JW, Crawford TW, Powell SB, Parker DE (1995). Compulsions in adults with mental retardation: Prevalence, phenomenology, and comorbidity with stereotypy and self-injury. American Journal on Mental Retardation.

[CR7] Burbidge C, Oliver C, Moss J, Arron K, Berg K, Furniss F, Woodcock K (2010). The association between repetitive behaviours, impulsivity and hyperactivity in people with intellectual disability. Journal of Intellectual Disability Research.

[CR8] Collacott RA, Cooper S-A, Branford D, McGrother C (1998). Epidemiology of self-injurious behaviour in adults with learning disabilities. The British Journal of Psychiatry.

[CR9] Cooper SA, Smiley E, Allan LM, Jackson A, Finlayson J, Mantry D, Morrison J (2009). Adults with intellectual disabilities: Prevalence, incidence and remission of self-injurious behaviour, and related factors. Journal of Intellectual Disability Research.

[CR10] Cooper SA, Smiley E, Jackson A, Finlayson J, Allan L, Mantry D, Morrison J (2009). Adults with intellectual disabilities: Prevalence, incidence and remission of aggressive behaviour and related factors. Journal of Intellectual Disability Research.

[CR11] Crawford DC, Acuna JM, Sherman SL (2001). FMR1 and the fragile X syndrome: Human genome epidemiology review. Genetics in Medicine.

[CR12] Crawford H, Moss J, Stinton C, Singla G, Oliver C (2018). Overactivity, impulsivity and repetitive behaviour in males with fragile X syndrome: Contrasting developmental trajectories in those with and without elevated autism symptoms. Journal of Intellectual Disability Research.

[CR13] Davies L, Oliver C (2013). The age related prevalence of aggression and self-injury in persons with an intellectual disability: A review. Research in Developmental Disabilities.

[CR14] Davies L, Oliver C (2016). Self-injury, aggression and destruction in children with severe intellectual disability: Incidence, persistence and novel, predictive behavioural risk markers. Research in Developmental Disabilities.

[CR15] Emerson E, Kiernan C, Alborz A, Reeves D, Mason H, Swarbrick R, Hatton C (2001). The prevalence of challenging behaviors: A total population study. Research in Developmental Disabilities.

[CR16] Farzin F, Perry H, Hessl D, Loesch D, Cohen J, Bacalman S, Hagerman R (2006). Autism spectrum disorders and attention-deficit/hyperactivity disorder in boys with the fragile X premutation. Journal of Developmental and Behavioral Pediatrics.

[CR17] Hatton DD, Hooper SR, Bailey DB, Skinner ML, Sullivan KM, Wheeler A (2002). Problem behavior in boys with fragile X syndrome. American Journal of Medical Genetics.

[CR18] Hessl D, Tassone F, Cordeiro L, Koldewyn K, McCormick C, Green C, Hagerman R (2008). Brief report: Aggression and stereotypic behavior in males with fragile X syndrome—Moderating secondary genes in a “single gene” disorder. Journal of Autism and Developmental Disorders.

[CR19] Hooper SR, Hatton D, Sideris J, Sullivan K, Hammer J, Schaaf J, Bailey DB (2008). Executive functions in young males with fragile X syndrome in comparison to mental age-matched controls: Baseline findings from a longitudinal study. Neuropsychology.

[CR20] Hyman P, Oliver C, Hall S (2002). Self-injurious behaviour, self-restraint, and compulsive behaviours in Cornelia de Lange syndrome. American Journal on Mental Retardation.

[CR21] Kushlick A, Blunden R, Cox C (1973). A method of rating behaviour characteristics for use in large scale surveys of mental handicap. Psychological Medicine.

[CR22] Le Couteur A, Rutter M, Lord C, Rios P, Robertson S, Holdgrafer M, McLennan J (1989). Autism diagnostic interview: A standardized investigator-based instrument. Journal of Autism and Developmental Disorders.

[CR23] Lord C, Rutter M, DiLavore P, Risi S (1999). Autism diagnostic observation schedule: Manual.

[CR24] McClintock K, Hall S, Oliver C (2003). Risk markers associated with challenging behaviours in people with intellectual disabilities: A meta-analytic study. Journal of Intellectual Disability Research.

[CR25] McDuffie A, Abbeduto L, Lewis P, Kim JS, Weber A, Brown WT (2010). Autism spectrum disorder in children and adolescents with fragile X syndrome: Within syndrome differences and age-related changes. American Journal on Intellectual and Developmental Disabilities.

[CR26] Munir F, Cornish K, Wilding J (2000). A neuropsychological profile of attention deficits in young males with fragile X syndrome. Neuropsychologia.

[CR27] Oliver C, Murphy GH, Corbett JA (1987). Self-injurious behaviour in people with mental handicap: A total population study. Journal of Intellectual Disability Research.

[CR28] Oliver C, Petty J, Ruddick L, Bacarese-Hamilton M (2012). The association between repetitive, self-injurious and aggressive behavior in children with severe intellectual disability. Journal of Autism and Developmental Disorders.

[CR29] Oliver C, Richards C (2015). Practitioner review: Self-injurious behaviour in children with developmental delay. Journal of Child Psychology and Psychiatry.

[CR30] Palmer J, Jenkins J (1982). The ‘Wessex’ behaviour rating system for mentally handicapped people: Reliability study. The British Journal of Mental Subnormality.

[CR31] Richards C, Jones C, Groves L, Moss J, Oliver C (2015). Prevalence of autism spectrum disorder phenomenology in genetic disorders: A systematic review and meta-analysis. The Lancet Psychiatry.

[CR32] Richards C, Moss J, Nelson L, Oliver C (2016). Persistence of self-injurious behaviour in autism spectrum disorder over 3 years: A prospective cohort study of risk markers. Journal of Neurodevelopmental Disorders.

[CR33] Richards C, Oliver C, Nelson L, Moss J (2012). Self-injurious behaviour in individuals with autism spectrum disorder and intellectual disability. Journal of Intellectual Disability Research.

[CR34] Rojahn J, Matson JL, Naglieri JA, Mayville E (2004). Relationships between psychiatric conditions and behavior problems among adults with mental retardation. American Journal on Mental Retardation.

[CR35] Rutter M, Bailey A, Lord C (2003). The social communication questionnaire.

[CR36] Smith LE, Barker ET, Seltzer MM, Abbeduto L, Greenberg JS (2012). Behavioral phenotype of fragile X syndrome in adolescence and adulthood. American Journal on Intellectual and Developmental Disabilities.

[CR37] Sullivan K, Hatton D, Hammer J, Sideris J, Hooper S, Ornstein P, Bailey D (2006). ADHD symptoms in children with FXS. American Journal of Medical Genetics Part A.

[CR38] Symons FJ, Clark RD, Hatton DD, Skinner M, Bailey DB (2003). Self-injurious behavior in young boys with fragile X syndrome. American Journal of Medical Genetics Part A.

[CR39] Taylor L, Oliver C, Murphy G (2011). The chronicity of self-injurious behaviour: A long-term follow-up of a total population study. Journal of Applied Research in Intellectual Disabilities.

[CR40] Thurman AJ, McDuffie A, Kover ST, Hagerman RJ, Abbeduto L (2015). Autism symptomatology in boys with fragile X syndrome: A cross sectional developmental trajectories comparison with nonsyndromic autism spectrum disorder. Journal of Autism and Developmental Disorders.

[CR41] Wilde L, Wade K, Eden K, Moss J, de Vries PJ, Oliver C (2018). Persistence of self-injury, aggression and property destruction in children and adults with tuberous sclerosis complex. Journal of Intellectual Disability Research.

